# Targeting the Choroid Plexuses for Protein Drug Delivery

**DOI:** 10.3390/pharmaceutics12100963

**Published:** 2020-10-14

**Authors:** Mark A. Bryniarski, Tianjing Ren, Abbas R. Rizvi, Anthony M. Snyder, Marilyn E. Morris

**Affiliations:** Department of Pharmaceutical Sciences, School of Pharmacy and Pharmaceutical Sciences, University at Buffalo: 304 Pharmacy Building, Buffalo, NY 14214, USA; mb247@buffalo.edu (M.A.B.); tren@buffalo.edu (T.R.); arrizvi@buffalo.edu (A.R.R.); snyder25@buffalo.edu (A.M.S.)

**Keywords:** choroid plexus, transcytosis, protein therapeutic

## Abstract

Delivery of therapeutic agents to the central nervous system is challenged by the barriers in place to regulate brain homeostasis. This is especially true for protein therapeutics. Targeting the barrier formed by the choroid plexuses at the interfaces of the systemic circulation and ventricular system may be a surrogate brain delivery strategy to circumvent the blood-brain barrier. Heterogenous cell populations located at the choroid plexuses provide diverse functions in regulating the exchange of material within the ventricular space. Receptor-mediated transcytosis may be a promising mechanism to deliver protein therapeutics across the tight junctions formed by choroid plexus epithelial cells. However, cerebrospinal fluid flow and other barriers formed by ependymal cells and perivascular spaces should also be considered for evaluation of protein therapeutic disposition. Various preclinical methods have been applied to delineate protein transport across the choroid plexuses, including imaging strategies, ventriculocisternal perfusions, and primary choroid plexus epithelial cell models. When used in combination with simultaneous measures of cerebrospinal fluid dynamics, they can yield important insight into pharmacokinetic properties within the brain. This review aims to provide an overview of the choroid plexuses and ventricular system to address their function as a barrier to pharmaceutical interventions and relevance for central nervous system drug delivery of protein therapeutics. Protein therapeutics targeting the ventricular system may provide new approaches in treating central nervous system diseases.

## 1. Introduction

The central nervous system (CNS) is protected by various barriers that are pivotal for maintaining a homeostatic environment for brain functions. One chief example is the blood-brain barrier (BBB) that sheaths the majority of cerebral blood vessels. Another is the barrier formed by the choroid plexuses (CPs) at the interfaces of the systemic circulation and ventricular system, comprising one of the blood-cerebrospinal fluid (CSF) barriers (BCSFBs). Though crucial for the protection of physiological functions in the brain, restricted CNS entry represents an obstacle for the delivery of therapeutic agents to treat CNS disorders. In this review, we will provide an overview of the CPs and ventricular system, including their function as a barrier to pharmaceutical interventions and relevance for CNS drug delivery of protein therapeutics.

## 2. The Structure and Cellular Organization of the Ventricular System

**Figure 1:**
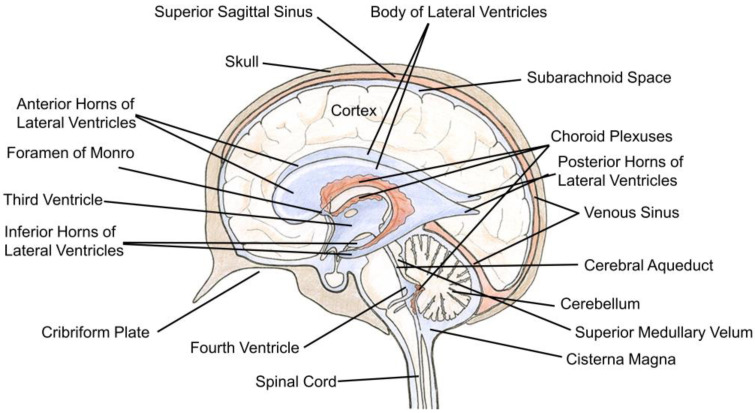
Anatomy of the brain and ventricular system. Image depicting the location of a number of structures covered within the current review. Cerebrospinal fluid is indicated by a light blue and the choroid plexuses are represented with a shade of red. Adapted with permission from [35], American Chemical Society, 2013.

The cerebral ventricular system is an interconnected succession of spaces that resides within the center of the brain. It is composed of four fluid-filled cavities: the third, fourth, and lateral ventricles (Figure 1). Each lateral ventricle exists and expands throughout one cerebral hemisphere. In either hemisphere, the lateral ventricle contains a central part, or body, located in the parietal lobe, an anterior horn located in the frontal lobe, a posterior horn located in the occipital lobe, and an inferior horn located in the temporal lobe. Separating the two lateral ventricles is the septum pellucidum that consists of a thin sheet of nervous tissue surrounded by ependyma. The lateral ventricles are connected to the third ventricle via the foramina of Monro (also known as the interventricular foramina). The third ventricle is located posteroinferior to the lateral ventricles and between the two thalami. It consists of five recesses: the infundibular, optic, anterior, pineal, and suprapineal recesses. These allow the third ventricle to branch off into surrounding tissue. Fluid passes from the third ventricle to the fourth ventricle via the Sylvian aqueduct (cerebral aqueduct). The fourth ventricle is located posteroinferior to the third ventricle in the hindbrain and is surrounded by segments of the pons, medulla, and cerebellum. It also contains five recesses; two lateral recesses, two lateral dorsal recesses, and a medial dorsal recess. CSF-filled spaces are present within the spinal column, but this is not a topic of discussion for the current review [1,2].

The ependyma is a single layered epithelium that coats the surface of the ventricular system. Its extent spans the lateral ventricles to the filum terminale of the spinal cord [3,4,5]. The ependyma is an intricate barrier that exhibits regional differences, heterogenous cell populations, and diverse functions whose complexities were described in an excellent introductory overview by Gabrion et al. in 1998 [6]. Ependymal cells that line the ventricular walls are cuboidal/columnar shaped and possess various organizations of cilia (uniciliated, biciliated, multiciliated) in addition to short microvilli on their apical surfaces [5,7,8,9]. Ependymal cells reside directly adjacent to the neuropil with various reports indicating the absence of a basement membrane [7,9,10]. However, basolateral labyrinths have been noted that can trap ventricular fluid and which purportedly connect to the perivascular spaces of the underlying ependymal vasculature [11,12,13,14]. Ependymal cells do not contain tight junctions between neighboring cells. Rather, gap junctions and zonulae adherins [5,15,16,17,18] facilitate the exchange of solutes and even macromolecules between the ventricular space and brain tissue [17,19,20].

Specialized ependymal cells are present throughout the ventricular system. Tanycytes are one example, which reside within the circumventricular organs (CVOs) bordering the third and fourth ventricles. CVOs are important homeostatic and communication hubs located in the CNS. There are several CVOs: the pineal gland, subcommissural organ, median eminence, vascular organ of the lamina terminalis, and the subfornical organ found within the third ventricle walls in addition to the area postrema located adjacent to the fourth ventricle. All but the subcommissural organ are vascularized by fenestrated capillaries that lack a BBB. They are typically classified by their function as either secretory or sensory CVOs [21,22,23,24]. Tanycytes are minimally ciliated ependymal cells with apical microvilli on their ventricular-facing membrane in addition to basal elongated processes. These projections penetrate the parenchyma and can contact blood vessels and neurons [4,10,25]. Tanycytes are classified into several different types based on a variety of factors that includes their location and morphology. They can possess tight junctions, but not always as was shown for tanycytes occupying the arcuate nucleus [26,27,28,29,30]. Tanycytes perform a variety of captivating and integral functions such as their role in neuroendocrine and metabolic regulation [25,31,32,33,34].

**Figure 2:**
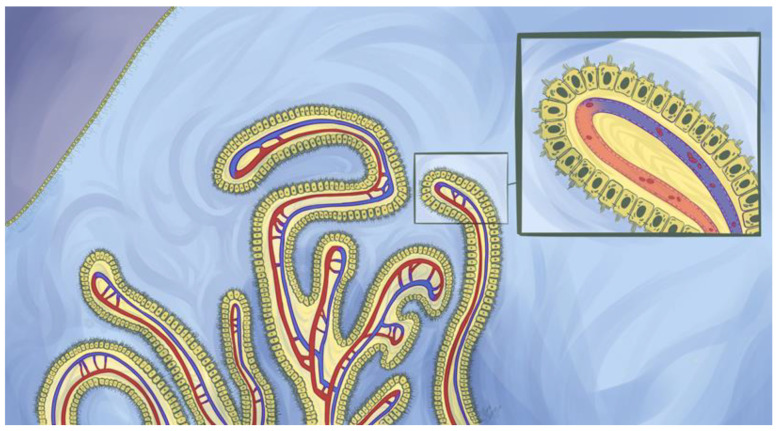
A choroid plexus suspended within a ventricle. The choroid plexuses are highly vascularized structures that house choroid plexus epithelial cells (CPECs). These cells are specialized ependymal cells that perform a variety of key functions within the central nervous system. CPECs are situated above fenestrated choroidal vessels that permit the rapid entry of fluid, solutes, and macromolecules which include endogenous and exogenous proteins (insert). However, tight junctions towards the paracellular apex between CPECs restrict the free exchange of material between blood and cerebrospinal fluid (CSF) and constitute a blood-CSF barrier. Two main paths have been proposed for protein flux across the CPECs: transcytosis following either fluid phase or receptor-mediated endocytosis and diffusion through the paracellular barrier. Additionally, CPECs possess developed microvilli and cilia on their apical membranes that are important instruments for CPECs to conduct their physiological functions. Above the depicted choroid plexuses are multiciliated ependymal cells that form a semipermeable barrier within the ventricular system. Brain parenchyma resides directly under the ependymal cell barrier and is depicted by a shade of purple. Ependymal cells play a pivotal role in regulating the movement of cerebrospinal fluid within the ventricular system.

**Figure 3:**
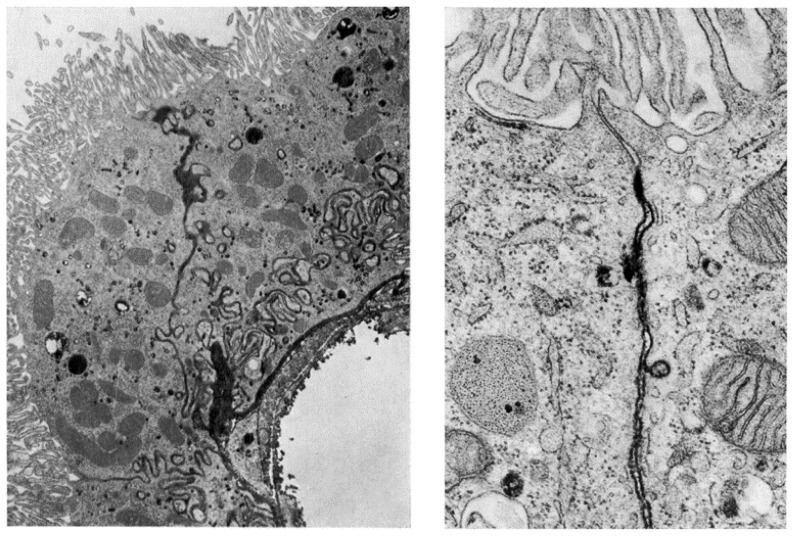
Electron microscopy images of the blood–cerebrospinal fluid barrier at the choroid plexus. An image captured 15 min after the intravascular administration of horseradish peroxidase (HRP, molecular weight ≈ 44 kDa). Seen are three choroid plexus epithelial cells (CPECs) with the apical membranes facing the ventricle at the top of the figure. The lumen of a fenestrated choroidal vessel can be seen at the bottom right. This image highlights the tight junctions between the CPECs via the dark black material (reaction product of the HRP), which can be found within the interstitial space at the basolateral membrane of the CPECs in addition to the paracellular/intercellular space between CPECs. Note the blockage of the HRP at the top of the paracellular tract just prior to the apical membrane. This is better visualized in B, where reaction product is visible along the entire paracellular space up to the point of the tight junction, at which it is stopped. A, ×13,000 magnification; B, ×73,000. Reproduced with permission from [19], Elsevier, 1968.

Another example of specialized ependymal cells is choroid plexus epithelial cells (CPECs). The CPs are tentacular tufts with a leaf-like organization suspended within every ventricle (Figure 1) [1,2]. Each appendage is composed of cuboidal CPECs anchored by an underlying stroma highly vascularized with fenestrated blood vessels (Figure 2) [19,36,37]. These vessels permit the rapid entry of fluid, solutes, and macromolecules into the interstitial interface between endothelial and CPECs as well as the paracellular spaces between adjoining CPECs (Figure 3). This feature is unlike parenchymal vessels which are tightly connected and bounded by the BBB [24]. However, the CP is not a permeable structure and constitutes one of the BCSFBs. Tight junctions seal adjacent CPECs and are composed of various transmembrane proteins that include zonula occludin-1 and members of the claudins [38]. On their apical membrane, CPECs possess more developed microvilli than those on ependymal cells [5,7,39]. This is an effective means to drastically increase the apical surface area for the CP, which is an important factor for transport processes [39]. In contrast, CPEC cilia are much smaller than those found on nearby ependymal cells which highlights the functional differences between these two cell types [5].

## 3. Functions of the Choroid Plexuses

The CPs perform various essential and complex functions that have been discussed by many prominent reviews [35,39,40,41,42,43,44,45,46,47,48,49,50]. Here, we will briefly highlight only a few roles beginning with the most recognized: the secretion of CSF. For an average human adult, there is around 90–150 mL of total CSF with approximately 20% found within the ventricular space [44,49,51,52,53]. About 0.5% of the total CSF volume is replaced by fresh fluid per minute in many mammalian species, with a production rate of ≈ 350–400 μL/min in humans [42,48,49]. Therefore, a volume corresponding to the total CSF volume in an adult human is produced and replaced roughly 4–5 times per day [48]. This high secretion capability is very dependent on the nature of the underlying choroidal blood vessels (fenestrations permit fluid flow) and the high rates of perfusion (3–5 mL/min/g tissue compared to the 0.35–0.4 mL/min/g in cerebral cortex) [42,49]. The majority of CSF is generated by the CPs, with other extra choroidal contributions coming predominantly from the brain interstitial fluid [48,52,54].

CSF content is actively regulated by the CPs, which contrasts with early misconceptions that CSF is a plasma ultrafiltrate [47,48,54]. CSF is almost entirely water with the remaining components including but not limited to ions, glucose, amino acids, peptides, proteins, and essential nutrients such as folate and vitamin C [47,53]. The CPs dictate the composition of the ventricular CSF through an intricate balance of biosynthesis, active transport, and efflux. For example, ascorbic acid is transported across the CPs by the sodium dependent vitamin C transporter (SVCT2) [35,53]. Studies have shown that SVCT2 is essential for maintaining brain ascorbic acid levels and SVCT2 knockout mice exhibit substantially reduced amounts of ascorbic acid in the brain [55]. Transthyretin (TTR) is produced by the CPECs where it plays a role in the transfer of thyroxine and retinol from the blood to the CSF [56,57]. Insulin is another example of a CP-produced peptide, which was recently demonstrated to be synthesized by CPECs and secreted in a serotonin-dependent manner [58]. These secreted proteins play important roles in overall CNS homeostasis [59].

However, not all CSF proteins are produced by the CPs. Serum albumin is a prime example of a plasma-derived protein that can cross the CPs and enter the ventricular space [60,61,62]. The CP–blood interface is an important barrier that plays a vital role in regulating the transport of molecules between blood and CSF. Like the BBB, various efflux and influx transport systems exist within CPECs to regulate the entry of endogenous and exogenous agents. For instance, members of the solute carrier (SLC) and ATP-binding cassette (ABC) families at the CPECs are involved in the bidirectional movement of small molecules, which have been discussed in our previous review [63] and other publications [64,65]. However, there is less information on the transport of peptides and proteins across the CPs, which may limit the future development of peptide/protein-based therapies for neurological diseases. Thus, the subsequent section will focus on the mechanisms of peptide and protein transport across the CPs (the combination of CPECs, stroma, vasculature, anatomical spaces, and cell-types not discussed here), including carrier-mediated transport for small peptides and receptor-mediated endocytosis and transcytosis for larger molecules.

## 4. Peptide and Protein Transport across the Choroid Plexuses

Peptide and protein movement from the blood to the CPECs is driven by anatomical arrangements and morphological characteristics. Upon reaching the choroidal vessels, serum proteins can rapidly traverse the CP vascular endothelium either by movement through endothelial fenestrae or, potentially, via vesicular shuttling. The endothelium is highly permeable to macromolecules such that even ferritin (≈474 kDa) can pass through the choroidal capillaries [19,36,50]. From here, protein will cross the endothelial basement membrane to arrive at the interstitial space between CPECs and endothelial cells. Proteins capable of diffusing through the CPEC basement membrane will then enter CSF via one of two likely paths. The first is paracellular convection between adjacent CPECs up to the point of the tight junction (Figure 2), followed by a slow diffusional “leak” through the apical tight junction into the CSF. This proposed mechanism is based on the inverse relationship between CSF/blood protein ratios with hydrodynamic radii and the structure of rat CP tight junctions, which were shown to be discontinuous such that proteins may be able to diffuse through [50,62,66]. However, this structural aspect has been noted to be challenging to confirm via ultrastructural studies [50] and would imply nearly all serum proteins, to some extent, should be present within CSF. Interestingly, a discontinuity in CPEC tight junctions of just 0.08% of the total perimeter would explain the trend between protein size and CSF/blood ratios [62,66].

The second potential route for proteins to enter the CSF is to undergo intact transcytosis across CPECs. This can occur following pinocytic uptake at the basolateral and intercellular membranes as shown for horseradish peroxidase (HRP, ≈44 kDa [19,67]), or via a specific receptor-mediated process as was shown for prolactin [68,69,70,71]. The expression and localization of several CPEC peptide and protein transporters, as well as their endogenous substrates and functions, are summarized in Table 1. The ensuing sections will detail various CPEC protein transport systems and their roles in serum protein entry into CSF.

### 4.1. Carrier-Mediated Transport

The transport of small peptides such as di- and tripeptides can be mediated through members of the proton-coupled oligopeptide transporter (POT) family (also known as the peptide transporter family) that belong to solute carrier proteins (SLC15A) [72,73]. Two peptide transporters from the POT family, PEPT1 (SLC15A1) and PEPT2 (SLC15A2), share similar substrates but have different affinities and localizations. PEPT1 is mainly confined to the small intestines whereas PEPT2 has a broader tissue distribution, including the CPs [72]. The mRNA expression of PEPT2 has been reported in the rat CPECs in early studies [74,75]. Later, in immunoblot and immunocytochemistry analyses, PEPT2 was found throughout the brain and the apical membrane of CPECs in both adult and neonatal rats but PEPT1 protein was absent from rat brain [76]. The direction of PEPT2-mediated transport has been studied in rat primary CPECs with glycylsarcosine as the model dipeptide, suggesting the role of PEPT2 in removing peptides from CSF to blood [77].

Amyloid-beta (Aβ) is a heterogeneous mixture of 37–43 amino acids and its accumulation in the brain is considered a major underlying mechanism for the development and progression of Alzheimer’s disease [78]. In addition to the flow out of brain parenchyma across the BBB, CSF bulk flow and CPEC-mediated removal have been implicated in eliminating Aβ from the brain [79]. The efflux transporter P-glycoprotein (Pgp/MDR1) on CPECs may play a role in this process [80]. Pgp is located at the apical side of human CPECs and is responsible for conferring an apical efflux of its substrates [81]. The mRNA and protein expression of Pgp at the CPEC has also been found in humans, mice, and rats [82]. Lam et al. illustrated the in vitro binding of human Aβ1-40 and Aβ1-42 peptides to hamster mdr1-enriched vesicles, suggesting Aβ is a substrate of Pgp [80]. Further studies supported the function of Pgp as an efflux pump for Aβ, implicating Pgp as a potential target for Alzheimer’s disease [80,83]. However, these in vitro studies were conducted on human kidney HEK293 and lung carcinoma LLC cells, which do not reflect the cellular composition of CPECs. More direct evidence for the role of Pgp in mediating Aβ transport at the CPEC is required.

### 4.2. Receptor-Mediated Transport

#### 4.2.1. Transferrin Receptor

The transferrin receptor (TfR) is a transmembrane glycoprotein that mediates the transport of iron-containing transferrin (Tf) [84]. The TfR has been found to regulate the supply of iron via receptor-mediated-endocytosis in a wide range of tissues. In most cells, after binding of the iron-Tf complex to TfR at the cell membrane, the complex is internalized through endocytosis, followed by endosome formation and acidification. Since iron-Tf binding is pH-dependent, iron is then released upon acidification and is transported through the endosomal membrane into the cytosol. The TfR bound with free Tf is then recycled to the cell membrane and released.

TfR in the CNS has been shown to regulate the transport of iron across brain barriers and maintain iron homeostasis in CPECs, brain capillary endothelial cells, and neurons [85,86]. Since TfR is highly expressed in the CPECs of both rats and humans, this site may play an essential role in the maintenance of iron homeostasis in the brain microenvironment [87,88]. Previous immunohistochemical analyses demonstrated the presence of TfR on the CPECs of rats [85], mice [86], and humans in the absence or presence of Restless Legs Syndrome [89]. Although Restless Legs Syndrome is a neurological disorder that may be attributed to CNS iron deficiency, the expression of TfR in afflicted patients was increased in the CPECs and decreased in brain microvasculature, suggesting a different function of TfR at the CPECs and BBB [89]. Wang et al. identified that TfR was localized around nuclei with large, discrete structures in rat CPECs and observed the movement of TfR to the apical cytoplasm after exposure to manganese or iron [90]. In addition to the TfR shift, the mRNA and protein expression of TfR was elevated after manganese exposure. The overexpression and translocation of TfR may be related to translational changes, but this requires further investigation. Deane et al. applied a brain perfusion technique in rats to demonstrate the role of CPECs in the rapid uptake of iron from the blood and subsequent release into CSF at a slower rate [91]. 

#### 4.2.2. Insulin and Insulin-Like Growth Factor Receptors

Insulin receptors are ubiquitously distributed in peripheral tissues where they play an essential role in regulating glucose homeostasis. In the brain, the insulin receptor differs in size, glycosylation, and insulin-binding affinity in comparison with the peripheral insulin receptor [92]. The in-situ hybridization study performed by Marks et al. revealed abundant mRNA of the insulin receptor in rat CPECs [93]. The high density of insulin receptors in CPECs was also reported in a quantitative analysis demonstrating the concentration of insulin binding sites in rat CPECs was equal to or greater than that in other brain regions or liver, which further suggested the CPs may be the target site for the transport of insulin from the blood into CSF [94,95]. However, the blood-CSF transport of insulin has not been directly investigated, including how this transport contributes to the insulin concentrations in brain interstitial fluid and how the differentiation between insulin-induced signaling and the purported transcytosis occurs within CPECs.

Besides the insulin receptor, insulin-like growth factor receptors (IGF1R and IGF2R) are involved in mediating the intracellular effects of insulin, insulin-like growth factor-I (IGF-I), and IGF-II [96]. Insulin receptor and IGF1R share similar structure but with different affinities to insulin and IGFs, whereas IGF2R is structurally dissimilar and binds only to IGFs but not insulin. IGF1R and IGF2R are expressed in the CPECs based on mRNA transcripts reported in rats [97,98,99,100]. Like the insulin receptor, a high density of IGF1R is presented in the CPECs in all ventricles in both rats and humans [95,101]. Nilsson et al. further conducted a study in pig CPECs and demonstrated a large number of IGF1R on the cell surface whereas IGF2R was distributed intracellularly [102]. In addition to CPECs, IGF2R was also found in the fenestrated capillary endothelial cells on BCSFB in infant rats [103]. IGF1R has been suggested to mediate the effect of IGF-I and IGF-II in CPECs while the role of IGF2R remains to be investigated [102]. Moreover, IGF1R also interacted with the low-density lipoprotein receptor-related protein 2 (LRP2)/megalin to transport IGF-I from blood to CSF [104].

#### 4.2.3. The Low-Density Lipoprotein Receptor Family

The low-density lipoprotein (LDL) receptor family comprises a group of plasma membrane receptors that play important roles in lipid metabolism and other physiological activities [105]. They are responsible for receptor-mediated transport of various ligands, including LDL, apolipoprotein E (ApoE), and other cellular nutrients, vitamins, and hormones. Several members also participate in pre- and postdevelopmental functions in the brain and may serve key roles in the pathogenesis of neurological diseases such as Alzheimer’s disease [106]. They share similar structures with ligand binding-type repeat domains and epidermal growth factor (EGF)-precursor homology domains at extracellular sites and at least one NPxY motif at an intracellular site for protein interaction and signal transduction [106].

Several core members of the LDL receptor family have been identified at the CPs, including the LDL receptor (LDLR), LDL receptor-related protein 1 (LRP1/LRP/α2-macroglobulin receptor), LRP2/megalin/glycoprotein 330, and LRP8/ApoE receptor 2 [107]. LDLR is the founding member of this receptor family and binds to cholesterol-rich LDL to mediate cholesterol uptake through clathrin-mediated endocytosis [106]. Matsumoto et al. performed an immunohistochemical analysis in seven autopsied human brain biopsies and demonstrated the distribution for LDLR in CPECs, with apical localization in some epithelium [79]. However, little is known on its expression and transport function at the CP and thus warrants further study.

Compared to LDLR, information on the role of LRP1 at the CP has been provided by additional studies. Previous work has examined LRP1 mRNA and protein expression in humans and rats [108,109,110,111,112]. An increase in the transcription of LRP1 at the rat CPECs was found with aging [109]. Fujiyoshi et al. performed a quantitative analysis via the use of liquid chromatography-tandem mass spectrometry and determined the content of LRP1 protein at rat CPECs to be 3.7 fmol/μg protein, which was much higher than the LRP2 protein amount (<0.20 fmol/μg protein) [111]. Intense immunohistochemical staining for LRP1 was found at rat CPECs, where a diffuse granular pattern was seen [113]. Although Matsumoto et al. showed negative immunostaining of LRP1 in human CPECs, Wolf et al. demonstrated clear staining for LRP1 in all analyzed brains [79,114]. The inconsistency may have resulted from the different antibodies and staining methods used in the two studies. LRP1 is also known as the α2-macroglobulin receptor as it could bind protease/α2-macroglobulin complexes and is involved in the clearance of the complex from CSF [35]. However, its transport mechanism through endocytosis or transcytosis requires further investigation. In addition to α2-macroglobulin, studies suggest that LRP1 may participate in the elimination of human Aβ (1–40) from the CSF, as suppression of LRP1 was associated with increased accumulation of intracellular Aβ in the CPECs [111,112,115]. During the Aβ elimination process, ApoE may also be involved by forming a complex with Aβ and then binding to LRP1 to promote the Aβ clearance via the CP [108].

LRP2, also known as megalin or glycoprotein 330, has been found at the apical and subapical site of CPECs in rats [109,116]. However, in the immunofluorescence study performed by Zheng et al., LRP2 staining was only found in apical and lateral sides of rat ependymal cells but not in CPECs [113]. Matsumoto’s work further demonstrated the presence of LRP2 immunostaining in both CPECs and human ependymal cells [79]. In contrast to the extensive expression of LRP1, the mRNA and protein expression of LRP2 at rat CPECs was low, with around 0.31 and 0.005, respectively, of its expression in rat kidney [117]. Decreased transcription and protein amounts of LRP2 were found in rat CPECs with aging, which may be associated with its role in Aβ transport at the CPECs [109]. CSF samples from patients with Alzheimer’s disease demonstrated reduced LRP1 and LRP2-bound Aβ, which may contribute to the elevated brain Aβ in Alzheimer’s disease [118]. LRP2 can bind to the ApoJ/Aβ complex to mediate clearance of the complex from CSF [119]. In addition to Aβ elimination, LRP2 can transport leptin and IGF-I from the peripheral circulation into the CSF across the CPECs [120,121]. Thus, it appears that the action of LRP2 at the CP is bidirectional, as it can transport solutes into the CPECs from both blood and CSF, followed by either degradation or transcytosis of the solutes. However, more comprehensive mechanistic studies need to be conducted to understand the functional activity of LRP2 at the CP.

LRP8 is also known as ApoE receptor 2 due to its function in the uptake of ApoE phospholipid discoidal particles or ApoE-enriched high-density lipoprotein [122]. It has been detected in the CPECs in rats and mice with mRNA expression displayed in rats [122,123]. In the brain, ApoE is synthesized and secreted by astrocytes and enriched in CSF, where ApoE may be taken up by CPECs by receptor-mediated endocytosis [122,124]. Selenoprotein P (Sepp1), a selenium-rich protein for supplying selenium to tissues including brain, may also be the substrate of LRP8 as the deletion of the LRP8 gene in mice resulted in undetectable levels of Sepp1 in the brain [123]. It is proposed that Sepp1 is taken up by LRP8 at CPECs from blood and free selenium or Sepp1 is then secreted into CSF [123].

#### 4.2.4. Neonatal Fc Receptor

The neonatal Fc receptor (FcRn) is a crucial facilitator of Immunoglobulin G (IgG) transport from mother to offspring, providing immunity to the newborn [125]. In addition to IgG, FcRn also interacts with albumin. It is responsible for rescuing IgG and albumin from intracellular degradation and mediating their bidirectional transport across different barriers [125]. The expression of FcRn has been found in CPECs in previous reports. Schlachetzki et al. presented the immunofluorescent staining of FcRn under confocal microscopy at rat CPECs demonstrating a diffuse pattern [126]. However, the reported cellular expression may be due to an artifact of acetone fixation since the Glut1 glucose transporter, which should be present on the basolateral membrane at the CPECs, was also found to exhibit a diffuse pattern in the cytoplasm in the study [126,127]. Latvala et al. further demonstrated the immunohistochemical staining of FcRn at the CP of cynomolgus monkeys, rats, wild type mice, SCID mice, and humanized transgenic mice expressing human FcRn (Tg32 and Tg276) [128]. The developing rat CP also demonstrated homogenous immunostaining of FcRn in epithelial cells and revealed predominant mRNA expression in developing stages compared with adults [35]. An in vitro transport study performed in polarized rat CPECs revealed the unidirectional transport of IgG from the CSF-facing side to the blood-facing side [35]. However, detailed evidence on the involvement of FcRn in IgG transport across the CP is currently lacking.

#### 4.2.5. SPARC

The SPARC (secreted protein acidic and rich in cysteine) is a soluble and cell surface albumin-binding glycoprotein that has been implicated in albumin transport from blood to CSF [129]. Higher immunostaining intensity of SPARC was found in *Monodelphis* and mice at very early age, which may be correlated with the higher protein concentrations in fetal CSF compared with adult CSF [129,130]. The basolateral membrane localization of SPARC in albumin-positive CPECs further suggested that SPARC can bind with albumin and act as a shuttle to transfer albumin from blood to CSF [129]. However, the relationship between SPARC and albumin is not linear and other molecular mechanisms affecting albumin binding and distribution may be involved, especially at older ages [130]. Thus, the current data only suggest the involvement of SPARC-mediated transport of albumin across BCSFB at early postnatal ages.

## 5. Preclinical Methodology to Study Protein Transport across the CPs

A variety of methods have been utilized to study protein transport across the CPs. A straightforward, yet indirect example is to administer a labeled or exogenous protein of interest systemically (e.g., intravenously) and measure CSF appearance over time. However, without supporting information such as time-dependent localization studies, this strategy does not directly implicate the CPs as the source of entry into the CSF. Here, we highlight several techniques to examine CP protein handling.

### 5.1. Imaging

Though not fully qualitative, imaging studies can provide crucial insight into the role of CPECs in the bidirectional transport of proteins. Early ultrastructural-level investigations examined the passage of HRP across the CPEC barrier from blood to CSF, and vice versa. These detailed investigations were instrumental in establishing the barrier properties of the CPs [19]. Other early imaging work demonstrated intraventricularly administered proteins accumulated on the CP stromal side [131,132], implying flux through the CPECs. Magnetic resonance imaging is a powerful technique that can provide crucial insight into ventricular physiology and drug distribution without invasive surgery, such as the recent evaluation of CP function [133]. Unfortunately, the depth of the CPs currently prevents the use of high resolution intravital two photon microscopy that has been used to study events occurring near the surface of the brain [134]. Therefore, many imaging strategies remain labor-intensive with poor temporal resolution because they require dedicated tissues for each group and time point. Furthermore, immunostaining is obligatory if using an unlabeled compound which can become burdensome when multiple study groups are present (e.g., multi-dose). Therefore, CP imaging should be utilized as a supporting technique in conjunction with more quantitative kinetic studies to fully characterize protein transport across the CPs.

### 5.2. Ventriculocisternal Perfusions

Ventriculocisternal perfusions have been employed for decades and were instrumental in delineating the physiology and dynamics of the ventricular system. These experiments typically encompass cannulations of one or both lateral ventricles and the cisterna magna for inflow and outflow, respectively [49,135,136,137]. Following a constant infusion of artificial CSF, test compound plus tracer can be infused intraventricularly and timed collections made at the outflow cannula. The tracer must be an inert substance that exhibits limited parenchymal penetration and metabolism over the study duration, and it can be used as the experimental standard. Repeated sampling at the outflow can facilitate the fraction of inflow extracted by the ventricular system. This data can be supported by a variety of techniques such as simultaneous blood sampling or autoradiographic/fluorescent imaging at various time points, depending on the label. As stated by Redzic and Segal, a clear benefit of this method is that it is performed in vivo and provides quantitative, kinetic information [49]. However, it is limited because it cannot truly detect CSF to blood flux across the CPs since the infused molecule/protein can distribute to other areas of the ventricular system (i.e., ependymal cells), brain, or both.

### 5.3. In Situ Choroid Plexus Perfusion

Another model for the study of CP transport is the ovine in situ CP perfusion. This method was originally developed to examine the CP independently of the BBB in a large animal [136,138]. A modified version was subsequently developed which eventually incorporated a pair of labeled compounds: an inert tracer and the compound of interest [49,139,140]. Briefly, the sheep brain is removed, and vascular cannulations are placed within the internal carotid arteries distally to the anterior choroidal arteries and the great vein of Galen [49,138,140]. This facilitates control and collection of the inflow and outflow to the isolated CP. For transport studies, the test and reference compounds are perfused into the CP via the arterial ligation and choroidal uptake calculated from the timed venous sampling as the fraction lost from the arterial input. The addition of the reference compound can facilitate the determination of non-specific uptake. Additionally, performing the study with the presence of excess unlabeled protein can be used to determine receptor-mediated endocytosis, as was performed for leptin [141]. This method can provide a substantial amount of information about the uptake kinetics at the basolateral surface of the CPECs. It also exhibits high sensitivity such that low extraction fractions can be measured. The method is limited by the intensive surgical preparations, its in situ nature which dramatically alters the local environment around the isolated CP, and the inability to measure bidirectional transport across CPECs [49].

### 5.4. Choroid Plexus Epithelial Cell Culture

Isolation and culture of primary CPECs remains the most straightforward model to evaluate bidirectional peptide/protein transport across a CPEC monolayer. Primary CPECs have been isolated and cultured from several preclinical species in addition to human tissue with varying success [142,143,144]. Most often, rodent CPECs are utilized for in vitro transport studies on semipermeable membrane inserts which segregate apical and basolateral faces via distinct chambers. CPEC monolayer formation and integrity can be monitored through a combination of transepithelial/endothelial electrical resistance readings and flux measurements of an inert molecule such as mannitol. Time and concentration dependent studies can then be conducted under a tightly controlled experimental environment and can incorporate treatment groups on an as needed basis. Recently, a transgenic mouse was developed that can alleviate the issue of non-CPEC cellular contamination [145]. These animals constitutively generate the tdTomato fluorescent protein in transthyretin-expressing cells, which includes CPECs. If coupled with fluorescence-activated cell sorting, investigators could isolate primary CPECs with high purity.

Limitations of primary CPECs are that a good extraction method is required to obtain acceptable viability and purity (when not using transgenic animals), they are time-consuming to isolate, and they require fresh tissue which can present a challenge for CPECs from both preclinical species and humans [142]. Several CPEC cultures are currently available that address at least some of these drawbacks, especially the convenience of growth within the laboratory. The HIBCPP cell line was derived from a CP papilloma in a female patient and exhibits several properties of primary CPECs [146]. As emphasized by Redzic, HIBCPP cells can be utilized as a CPEC-like model to address specific investigational questions if cultured under explicit conditions [143,147]. This is due to limitations ranging from their tendency to stack when growing and a monolayer appearance atypical of the traditional cobblestone arrangement of primary CPECs [143]. Rat Z310 cells represent another CPEC culture utilized to study CP physiology and transport [148,149]. However, Z310 cells and the alternative CPEC models (including primary isolations) do not completely represent CPECs in vivo. For example, transporter expression was shown to be significantly different in Z310 cells when compared to primary rat CPECs [150]. Thus, CPEC culture models should always be used in conjunction with supporting studies to accurately define CPEC transport processes [49].

By combining complimentary techniques, investigators can delineate the transport of therapeutic proteins across the CPs into the CSF, the direction that is oftentimes the focus of drug delivery. A good example of such a study was the examination of apolipoprotein A-I entry into CSF [151]. Several experimental aspects should be considered when conducting these studies. First, groups examining CP protein transport and evaluating ventricular disposition should strongly consider the use of adult animals (if adult patients are targeted) for experiments and primary CPEC isolations due to the developmental changes in the ventricular system and CPs [18,152,153,154,155]. Second, transport studies of endogenous proteins should include a transcript-level examination to determine if CPECs can synthesize the protein/peptide of interest. A third factor is the potential for ventricle-specific transport differences due to regional CPEC variations. Previously, differential CPEC gene expression and secretory profiles were demonstrated to be spatially distinct between the lateral and fourth ventricles [152]. Examining the discrete contributions of each CP would provide important insight into regional differences but would entail, at the very least, detailed transcriptomic, proteomic, kinetic, and imaging analyses. Finally, CSF secretion rate and/or clearance should also be included in CP protein transport studies as it has been emphasized CSF dynamics can drive protein concentrations within the CSF (discussed in [156]). For example, increased CSF protein concentrations can be the result of reduced CSF flow rather than increased CP permeability. Therefore, such measurements would be especially crucial when examining the impact of disease state on endogenous/exogenous protein transport at the CPs, where alterations in CSF dynamics can influence experimental observations and thus conclusions.

## 6. Targeting the Choroid Plexuses for Brain Delivery: Futile Strategy or Deviously Wise?

CNS disorders represent a massive economic and healthcare burden on the global population. Numerous CNS diseases and injuries leave irreparable damage and remain difficult to impossible to treat. As well, in too many cases, current treatment options are only palliative and do not address the underlying pathologic drivers, if they are even known (see [157] for an excellent review on this topic). A key obstruction in CNS treatment is the difficulty in penetrating the CNS barriers to deliver therapeutic compounds and proteins at efficacious concentrations. The CPs and ventricular system have been targeted to deliver drugs to the parenchyma because of the ability to circumvent the BBB. However, as has been noted repeatedly, this may not be the best strategy to reach therapeutic targets deep within brain tissue [158,159]. In the ensuing sections, we will briefly discuss the fluid dynamics and biological barriers of the ventricular system and how they are important mediators of protein/peptide biodistribution. We will also highlight the potential utility of delivering protein therapeutics to the CSF for the treatment of CNS disorders. 

### 6.1. Intracranial Cerebrospinal Fluid Flow—Overview

As stated earlier, the majority of CSF production has been shown to be from the CPs. The net flow of CSF from the lateral ventricles occurs in the direction through the foramen of Monro into the third ventricle, which then makes its way through the Sylvian aqueduct to enter the fourth ventricle. From here, CSF will enter the subarachnoid space (SAS) via the foramina of Magendie and Luschka in a manner that CSF reflux from the SAS back into the fourth ventricle is typically associated with pathophysiology [49,160,161,162,163,164,165]. CSF within the intracranial SAS can then envelop the brain via travel within the leptomeninges, which is composed of the arachnoid and pia mater [24]. Though still indeterminate, CSF can exchange between the subarachnoid and subpial spaces due to the lack of well-developed junctional barriers within the pial membrane [166,167,168,169]. However, CSF and its contents cannot readily access the subjacent brain parenchyma due to the glia limitans composed of astrocytic end feet and the associated basement membrane [131,170].

From the subpial and subarachnoid spaces, CSF can also pass into perivascular spaces (PVSs) surrounding arterioles, capillaries, and likely venules within the brain. The PVS is a physical space that facilitates the distribution of fluid and solutes via bulk flow adjacent to brain vessels. It represents a site of CSF and brain interstitial fluid (ISF) exchange. PVS fluid movement is proposed to be highly driven by arterial pulsations [171,172,173,174]. Unfortunately, an exact anatomical description for the PVS remains contested and the reader is referred to other publications for further details [160,166,175,176,177]. Due to this uncertainty, the specific route CSF follows to enter the PVS is also unclear. What is known is that tracer administered intracisternally or intraventricularly can enter the PVS to distribute throughout the CNS, evidenced by numerous studies over the past several decades [134,166,171,178].

PVS fluid can enter the brain parenchyma, but the extent and movement remain hotly disputed and are an active field of neuroscience research. One hypothesis (the glymphatic system) proposes CSF inflows into the arterial PVS in the same direction as blood flow, driven by arterial pulsations as discussed directly above. CSF then enters the parenchyma through astrocytic end feet, which is heavily dependent on aquaporin-4 (AQP4) water channels. This influx of fluid facilitates mixing between CSF and brain ISF. Parenchymal exit occurs via the perivenous PVS and tracts located alongside cranial nerves (perineural tracts, see [12] for example). A tenet of the glymphatic system is the massive impact of sleep and awake states (see [175] for a recent glymphatic description). However, the intraparenchymal bulk flow incorporated within the glymphatic hypothesis directly conflicts with data indicating diffusion as the singular driver of fluid and solute movement through most of the brain extracellular space. The ongoing debate on fluid movement between and within the parenchyma and the PVS is not a topic for the current work and the reader is referred to a trove of information in numerous reviews [160,166,175,176,179,180,181,182,183,184,185]. Additionally, differential movement of fluid in white and gray matter [179] plus intraparenchymal drainage sites (e.g., extrachoroidal CSF sources, ventricular mixing, etc.) warrants a more detailed discussion than can be included here [12,186]. What is important for the current work is that substances within the PVS can enter the brain parenchyma, effectively bypassing the BBB, as well as leave the parenchyma and enter the PVS.

Intracranial CSF absorption has been shown to occur through two major pathways. The first is clearance from the SAS via arachnoid granulations, which act as unidirectional regulators to direct CSF into the venous circulation [187,188]. The second intracranial CSF exit route is lymphatic drainage [187,188,189,190,191]. CSF lymphatic outflow can occur via travel within the perineural space surrounding cranial nerves and subsequent lymphatic drainage. The nasal drainage route (perineural space alongside the olfactory nerves, through the cribriform plate, and eventual lymphatic drainage in nasal submucosa) has been demonstrated as an important path [192,193,194]. A recent investigation has also identified the role of basal meningeal lymphatics in clearing CSF [195]. Though both routes likely play a role in the clearance of protein therapeutics from the CSF, studies measuring the quantitative contributions of these CSF exit routes remain scarce (e.g., [193]) and the specific contributions are still poorly defined, especially in humans [196].

The paths of intracranial CSF flow through and out of the brain are not just important for CNS physiology and disease. CSF flow is a predominant factor for protein therapeutic distribution within the brain. Therefore, understanding how CSF moves within the brain would provide important predictive insight on spatial exposure: if the compound is delivered intraventricularly through the CPs, an anticipated scenario would be high ventricular, SAS, PVS, and perineural distribution with both lymphatic and venous clearance. These locations would experience higher exposure profiles when compared to regions further away from robust CSF flow. Furthermore, the rate of CSF clearance is an important mediator of overall brain exposure such that influx of the therapeutic payload through the CPs must exceed CSF turnover, or else efficacious concentrations may not be achieved [156,192,197]. These and other aspects must be taken into consideration when deciding if ventricular delivery across the CP barriers is the best option for the CNS target site [198].

### 6.2. Intracranial Barriers to Cerebrospinal Fluid-Brain Parenchyma Exchange of Protein Therapeutics

#### 6.2.1. The Choroid Plexuses

In addition to the transport of substances into the CSF, CPECs actively remove substances from the ventricles. This has been demonstrated using exogenous and endogenous compounds [39,41]. Past results indicated CPECs facilitate apical to basolateral (ventricle to blood) albumin transport in an energy-dependent manner [131,199]. Another study provided evidence that IgG undergoes unidirectional apical to basolateral (ventricular efflux) transport by cultured CPECs [35]. These results were consistent with negligible IgG CSF entry across the CPs [200]. However, it is still uncertain to what extent and by which cellular mechanisms CPECs efflux proteins. Opposing work has indicated a minimal CPEC contribution for the removal of ventricular albumin [61,132]. The work on IgG efflux requires further confirmation through a detailed in vivo study.

It also remains unclear how a CPEC protein transporter can facilitate influx without also conducting efflux of that same ligand. For example, CPEC megalin has been implicated in the transport of proteins into the CSF (e.g., leptin, IGF-1) [104,120,121] as well as efflux from the CSF (e.g., amyloid-beta-ApoJ complex) [201]. This would imply megalin is at least present within the basolateral and apical CPEC membranes, which suggests megalin could also conduct ligand binding within the ventricle to remove the same substances it purportedly transports into the CSF (i.e., leptin). Detailed, high resolution localization studies for CPEC megalin and many other protein transporters have yet to be conducted but work thus far indicates megalin is diffusely expressed throughout CPECs [79,109,121]. This contrasts with its typical apical localization within epithelial cells, such as proximal tubule epithelial cells where megalin is found in apical regions and to a lesser extent in lysosomes. In the kidneys, megalin facilitates ligand binding and internalization, with receptor recycling back to the apical membrane [202]. Therefore, it is unclear why megalin would perform apical reabsorption in the proximal tubules but basolateral and apical uptake in CPECs. Furthermore, megalin trafficking patterns in the proximal tubules indicate its ligands are directed towards lysosomal degradation if no salvage pathway is present. Alternative receptors are required for transcytosis, such as FcRn for albumin [203]. Overall, these questions emphasize the need for mechanistic and thorough localization/trafficking studies for megalin and many other protein transporters expressed in CPECs.

#### 6.2.2. The Ependymal Cells

CSF flow within the ventricular system is not constant and unidirectional. Rather, it is pulsatile and complex with macroscale and near-wall variations. Ependymal ciliary beating is a major driver of ventricular CSF bulk movement and directionality, with other major influences including cardiac pulsations and inspiration [204,205,206,207]. Additionally, ependymal cells do not possess tight junctions, which permits rapid fluid/content exchange of even macromolecules and proteins [17,20]. Together, these aspects are likely major factors that promote mixing between ventricular and parenchymal compartments.

Despite the lack of tight junctions, the ependymal cells should be considered a barrier to protein therapeutics administered intraventricularly or delivered across the CPs [5,198]. Ependymal cells express both specific and multiligand receptors that can induce target-mediated disposition or receptor-mediated endocytosis of a protein therapeutic. Past work has suggested the negligible entry of ventricular brain-derived neurotrophic factor (BDNF) into brain tissue was caused, in part, by high affinity BDNF-binding receptors expressed by ependymal cells [198,208]. A previous histochemical study provided evidence that several promiscuous receptors were present on ependymal cells, including LRP1 and megalin [79]. Even without ligand binding, the mechanical and physical aspects of the ependymal–parenchymal interface likely play a role in limiting protein therapeutic entry into the brain. For example, though a large protein can easily access the basolateral portion of ependymal cells, the physical characteristics of the brain extracellular space will restrict protein movement into the brain [179]. If subependymal channels do exist, as discussed earlier, these could act as reservoirs to hold ventricular contents [11]. These cellular and physical features would facilitate ependymal uptake and subsequent transport back into the ventricular space. Such ependymal facets may govern the fate of ventricular albumin, where ependymal fluid-phase and receptor-mediated endocytosis likely occurs. This is supported by the ependymal expression of two albumin transcytosis receptors: CD36 and FcRn (unpublished results, [79,125,209]), which imply frequent endocytosis of albumin. However, directionality, capacity, and kinetics of this transport is unstudied.

These statements should not be taken as an ultimatum that proteins cannot enter the brain parenchyma from the ventricular space. The rate and extent of CNS distribution for any compound are dependent on numerous factors. Here, we are emphasizing that the ependyma should be taken into consideration when determining/predicting the biodistribution of protein therapeutics. Similar to protein transport by CPECs, detailed studies are truly needed to better understand ependymal cell biology and their role in regulating the CNS disposition of therapeutic proteins.

#### 6.2.3. The Perivascular Space

As discussed above, the PVS is considered a linkage of CSF with SAS and ISF at the brain parenchyma [166,177]. Increasing evidence has illustrated that the PVS facilitates the distribution of CSF substances, including macromolecules, along its cerebrovascular tree to varying extents through the brain, such as full-length IgG and smaller single-domain antibodies after intrathecal infusions in rats [167] and conjugated albumin after intracisternal infusion in mice [210].

Be that as it may, barriers lie in this compartment to further restrict the exchange of compounds between CSF within the PVS and brain parenchyma. The pial surface is regarded as an important interface between the SAS and brain parenchyma [211]. During the early developing stage, the presumptive pia-arachnoid consists of large fenestrated sinusoid but can still restrict the entry of albumin as demonstrated in rat at embryonic day 12 [212]. A later study further observed the closely packed layer of leptomeningeal cells in the initial pial surface, which suggested that the pial surface contributes to diffusion restriction [211]. In addition to the pial membrane, the access of CSF substances to brain through the PVS pathway may be size-dependent. Iliff et al. observed that although 3 and 2000 kDa dextran are rapidly transported along the PVS after intracisternal injection in rats, the smaller dextran showed greater extent of distribution in brain interstitium while the larger dextran concentrated around the PVS [134]. Similarly, smaller single-domain antibodies demonstrated a greater area of distribution in brain slices than full-length IgG [167]. It was hypothesized that the sieving by stomata on leptomeningeal cells at subarachnoid vessels may contribute to the size-dependent access [167]. The PVS also showed scavenging functions to phagocytose substances to influence their brain disposition. A number of dense lysosomal bodies were presented in the perivascular cells under electron microscopy observation, which were distinct from pericytes [213]. Wagner et al. observed the rows of pinocytotic invaginations from the basement membrane of PVS and vesicles fusing with the luminal plasma endothelium after intraventricular administration of HRP, providing a pathway for removing protein from CSF to blood [178]. 

Other factors influencing the PVS distribution, such as physiochemical properties, state of consciousness, circadian rhythm, and diseases or surgical intervention, can significantly alter PVS physiology and thereby CNS distribution of protein therapeutics [166,167,210]. Thus, the PVS should be considered as another barrier in CSF targeting brain delivery. 

#### 6.2.4. Why Target the Choroid Plexus for Central Nervous System Protein Drug Delivery?

As the systemic or intraparenchymal delivery of protein therapeutics may be greatly hindered by BBB and brain tissue structure, targeting the CSF space, adjacent cerebral structure, or CPs may offer another approach in the treatment of CNS diseases.

First, the therapeutic targets may locate at subarachnoid, perivascular, and periventricular spaces and their adjacent tissues. In several neuroinflammatory disorders, such as experimental autoimmune encephalomyelitis [214], multiple sclerosis [215], and cytomegalovirus infection [216], the pathogenic lymphocyte, monocyte, and neutrophil cell populations may accumulate within CSF. These fluid-filled spaces also connect with deep cervical lymph nodes [216,217], which may serve as the targets of interest for therapeutic immunoregulators. In the case of ventricular tumors and lesions, delivery of therapeutics to their contact CSF spaces may benefit the treatment. Hypothalamic tanycytes, the CSF contacting cells located at the third ventricles [218], may also be a target for CNS therapeutics. Recent studies unraveled their function in regulating appetite and energy balance [33]. Thus, targeting these cells may modulate the metabolic and neuroendocrine functions to tackle diseases such as anorexia and hyperphagia. Additionally, CSF contacting neurons within the third ventricles may also be targeted due to their function as pH sensors and mechanoreceptors to regulate brain homeostasis [219].

Second, CSF circulation and PVS pathways may bring administered protein therapeutics to wider areas and deeper brain distributions. Intrathecal administration of protein therapeutics is regarded as a promising delivery method to bypass the BBB to reach brain parenchyma. Examples include the treatment of lysosomal storage disorder mucopolysaccharidosis by intrathecal delivery of recombinant human alpha-L-iduronidase or iduronate-2-sulfatase, which resulted in enzyme accessing the superficial and deep brain tissues in dog and mice [220,221]. The PVS as a route for CSF and ISF inter-exchange may also contribute to brain distribution in addition to CSF diffusion. However, further work that includes kinetic studies with longer duration remain needed. On the other hand, intraventricular delivery did not result in deep brain distribution, which may be restricted by the ependymal lining as discussed above [222]. Other delivery systems with permeability at the ependymal layer may be required.

As a medium to carry brain and blood-derived signals to distant targets within the brain, CSF should be taken into consideration for protein therapeutic brain delivery. However, the complex physiology of the subarachnoid, perivascular, and ventricular spaces in addition to the confounding factors influencing CSF flow in health and disease are still not entirely understood. Careful deliberation should thus be included in decision making when determining if CP-mediated delivery is the best option as the CNS delivery strategy.

## 7. Clinical Use of Peptide/Protein-Based Therapies for Neurological Diseases

**Figure 4:**
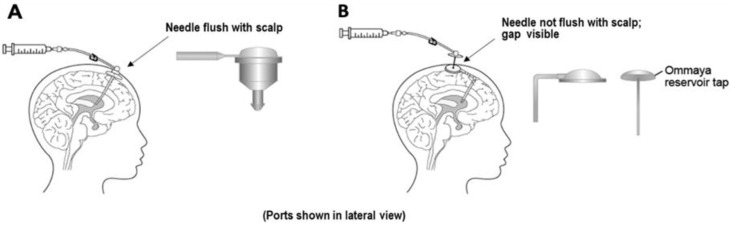
Illustration of intraventricular administration by (**A**) Rickham reservoir and (**B**) Ommaya reservoir. Reproduced with permission from [225], Elsevier, 2020.

Although peptide/protein-based therapies have been introduced in various indications, their application in CNS diseases remains challenging. Based on the summary of approved peptide/protein therapies for CNS diseases (Table 2), it was found that these therapies are mainly approved for treatment of multiple sclerosis and adult migraine with administration through intravenous and subcutaneous routes. One exceptional case is cerliponase alfa (Brineura™), a recombinant proenzyme form of human tripeptidyl peptidase-1 (TPP1) approved for treatment of neuronal ceroid lipofuscinosis type 2 (CLN2) disease via intraventricular infusion [223]. CLN2 is a pediatric neurodegenerative disease resulting from TPP1 deficiency, leading to increased lysosomal storage material that may cause a progressive decline in motor and language functions [224]. Administration of cerliponase alfa directly into ventricles can facilitate its distribution into brain to clear the lysosomal storage material and prevent the immune-mediated adverse effects caused by systemic enzyme-replacement therapy [223]. In the clinical study, cerliponase alfa was administered into the cerebral lateral ventricle with surgically implanted Ommaya or Rickham reservoirs (Figure 4) [223,225]. The C_max_ of cerliponase alfa in CSF was 1260, 1630, and 1390 µg/mL at day 1, week 5, and week 13 after intraventricular infusion, which resulted in CSF/plasma ratio of 1200, 809, and 1320, respectively [226]. The AUC_0–t_ in CSF was 393, 340, and 1330 times higher than that in plasma at day 1, week 5, and week 13 after administration. The results of this study indicated a high CNS distribution with limited systemic circulation of cerliponase alfa after intraventricular administration, which may contribute to the reduced decline in motor and language function compared with historical controls. However, serious adverse effects associated with the intraventricular device, including device-related infections and leakage, have been found during the clinical study, leading to interruption of the treatment [223]. 

Other potential peptide/protein therapies are under investigation for a wider range of neurological diseases, including Alzheimer’s disease, glioblastoma, and other brain tumors (Table 3). For the treatment of brain tumors, radioimmunotherapy is directly administered into the postsurgical resection cavity. An example is the administration of ^188^Re-labeled nimotuzumab and ^211^At-labeled 81C6 mAb, which enable efficient delivery of radiation doses to the tumor area and limit potential harm to the surrounding normal brain region and distant organs [227,228]. ^131^I-Omburtamab is a radioimmunotherapy agent that targets the glycoprotein B7-H3 expressed in neuroblastoma and is being assessed for treatment of metastatic leptomeningeal tumors [229]. In the clinical study, ^131^I-omburtamab was intraventricularly administered via the Ommaya shunt, which resulted in around 10-fold CSF-to-blood ratios based on PET analysis and generally low radiation doses to other organs besides brain parenchyma and liver [229]. Thus, intraventricular infusion promotes the exposure to the CSF spaces and leptomeninges while limiting systemic toxicity. However, high interindividual variation in CSF exposure was found, which may be attributed to the dose retained in the lateral ventricle, likely related to disease [229].

Overall, cerliponase alfa and ^131^I-omburtamab are examples for clinical use of peptide/protein therapy targeting CPs, which maximize brain distribution while minimizing systemic side effects. The drawbacks of this delivery method include device-related complications and variability in ventricular distribution.

## 8. Conclusions

It is clear from the failures to develop CNS drugs that a better understanding of CNS physiology and fundamental cellular biology is desperately needed. Various CNS barriers are anatomically positioned to obstruct the penetration of therapeutic compounds and proteins at efficacious concentrations. Although targeting the CPs and ventricular system may be an option to deliver drugs to brain parenchyma without impairment by the BBB, other factors deserve attention for this delivery pathway, including CSF fluid dynamics, CPECs, ependymal cells, and PVSs. During the experimental examination of CP delivery, physiological conditions should always be closely monitored as change in CSF dynamics can alter the therapeutic disposition. Due to the developmental changes in CPs and the ventricular system, the experimental model should correspond to the age of the targeted patient population to provide better correlations. Studies on carrier/receptor-mediated transport at CPECs have paved the way for new pathways of peptide and protein therapy delivery. However, further elucidation of the receptor–ligand co-localization and trafficking studies are warranted to provide direct evidence in CPEC transport systems. Further development of inducible knockout models targeting CPEC transporters are needed to better understand CP protein handling. With a better understanding of the physiological processes and comprehensive experimental techniques, the CPs have the potential to be targeted as a promising approach to treat specific CNS disorders.

## Figures and Tables

**Table 1:** Summary of transporters and receptors involved in transport of peptides and proteins at blood–cerebrospinal fluid (CSF) barriers (BCSFB).

Transporter/Receptors		Expression	Localization	Endogenous Substrate	Direction	Function
Peptide transporter (PTR) family	PEPT2/SLC15A2	mRNA (r) [74,75]	A of CPECs (r) [76]	Di-/tripeptide [73]	Efflux, CSF→luminal [76]	Removal of neuropeptides, peptide fragments, and peptide-like drugs from CSF [76,77]
P-glycoprotein	Pgp/MDR1	mRNA and protein (r, m h) [82]	A of CPECs (h) [81]	Aβ [80,83]	Efflux, CSF→luminal [80,83]	-
Transferrin (Tf) receptor	TfR	mRNA and protein (r) [90]	CPECs (h) [89], (r) [85], (m) [86], vesicles around nuclei of CPECs (r) [90]	Tf [86,91]	Unidirectional uptake (luminal→CP epithelium) [91]	Uptake of Tf bound iron into CPECs and slow release of iron to CSF [91]
Insulin receptor		mRNA (r) [93]	CPECs (r) [94,95]	Insulin [94]	Luminal→CSF [94]	May transport insulin from the blood into the CSF with intermediate compartment and saturable process [94]
Insulin-like growth factor receptors	IGF1R	mRNA (r) [97], (r fetus) [98]	CPECs (r) [95], (h) [101], on surface of CPECs (p) [102]	IGF-I, IGF-II, insulin [96]	Luminal→CSF [104]	Mediates effects of IGF-I and IGF-II [102]
	IGF2R	mRNA (r) [99,100]	Intracellular of CPECs (p) [102], epithelium and endothelium CP (infant r) [103]	IGF-I, IGF-II [96]	-	-
Low density lipoprotein (LDL) receptor	LDLR	-	A of CPECs (h) [79]	LDL [107]	-	-
LDL receptor-related proteins (LRPs)	LRP1 (LRP/α2-macroglobulin receptor)	mRNA and protein (h) [108,112], (r) [109], mRNA (r) [110], protein (r) [111]	Diffuse cellular of CPECs (r) [113], CPECs (h) [79]	α2-macroglobulin [35], Aβ [35,111,112]	Efflux (CSF→luminal) [109]	May involve in the clearance of protease/α2-macroglobulin complexes from the CSF [35] Maintains brain homeostasis of Aβand partly mediates the elimination of Aβ from CSF [111,112] May associate with apolipoprotein E (apoE) to influence the severity of cerebral amyloid angiopathy and Alzheimer’s disease [108]
	LRP2 (megalin/glycoprotein 330)	mRNA and protein (r) [109,117]	A of CPECs (r) [109,116], CPECs and ventricular ependyma (h) [79]	Leptin [120], IGF-I [104,121], ApoJ [119]	Bidirectional transport [35]	Mediates entry of leptin into CSF across CP [120] Mediates penetration of peripheral IGF-I in the CSF and mediates IGF-I-induced clearance of Aβ [121] Bind with ApoJ and mediates clearance of Aβ1-40-apoJ from CSF [119]
	LRP8 (ApoE receptor 2)	mRNA (r) [122]	CPECs (r) [122], (m) [123]	ApoE [122], selenoprotein P (Sepp1) [123]	-	May involve in the uptake of ApoE phospholipid discoidal particles or ApoE-enriched high-density lipoprotein in brain [122] Facilitates uptake of Sepp1 [123]
Neonatal Fc receptor	FcRn	mRNA (r) [35]	Diffuse cellular of CPECs (r) [35,126], CPECs (monkey, r, m) [128]	IgG [126]	-	May mediate transcytosis of IgG [126]

CP: choroid plexus, b: basolateral side, a: abluminal/apical side, h: human, r: rats, m: mice, p: porcine, –: not reported.

**Table 2:** Approved peptide/protein-based therapies for neurological diseases.

Name	Brand Name	Description	Condition/Disease	Route of Administration	Initial Approval Year
Cerliponase alfa	Brineura™	Recombinant human tripeptidyl peptidase-1	Neuronal ceroid lipofuscinosis type 2 disease	i.c.v.	2017
Glatiramer acetate	Copaxone^®^	Acetate salts of synthetic polypeptides of L-glutamic acid, L-alanine, L-tyrosine, and L-lysine	Relapsing forms of multiple sclerosis	s.c.	1996
Peginterferon beta-1a	Plegridy™	Interferon beta-1a	Relapsing forms of multiple sclerosis	s.c.	2014
Natalizumab	TysabriI^®^	Humanized IgG4k monoclonal antibody	Relapsing forms of multiple sclerosis	i.v.	2004
Ocrelizumab	Ocrevus™	Humanized anti-CD20 monoclonal antibody	Relapsing or primary progressive forms of multiple sclerosis	i.v.	2017
Ofatumumab	Kesimpta^®^	Anti-CD20 monoclonal antibody	Relapsing forms of multiple sclerosis	s.c.	2020
Eptinezumab	Vyepti™	Humanized IgG1 antibody antagonizing CGRPR	Adult migraine	i.v.	2020
Erenumab	Aimovig™	Human monoclonal antibody antagonizing CGRPR	Adult migraine	s.c.	2018
Fremanezumab	Ajovy™	Humanized IgG2 antagonizing CGRPR	Adult migraine	s.c.	2018
Galcanezumab	Emgality™	Humanized IgG4 antagonizing CGRPR	Adult migraine	s.c.	2018
Dinutuximab	Unituxin™	GD2-binding monoclonal antibody	Pediatric patients with high-risk neuroblastoma	i.v.	2015

CGRPR: antibody antagonizing calcitonin gene-related peptide receptor; i.c.v.: intracerebroventricular; s.c.: subcutaneous; i.v.: intravenous.

**Table 3:** Peptide/protein-based therapies in clinical trials for neurological diseases.

Name	Description	Condition/Disease	Route of Administration	Status	Reference/Clinical Trial Identifier
Aducanumab	Human monoclonal antibody targeting Aβ	Alzheimer’s disease	i.v.	Phase III (under review)	NCT02477800; NCT02484547
Gantenerumab	Human IgG1 antibody targeting Aβ	Alzheimer’s disease	s.c.	Phase III	NCT01224106; NCT01760005; NCT02051608; NCT03443973; NCT03444870
ABBV-8E12	Humanized IgG4 anti-tau antibody	Alzheimer’s disease	i.v.	Phase II	NCT02880956
AL002	Anti-human TREM2 antibody	Alzheimer’s disease	i.v.	Phase I	NCT03635047
AL003	Anti-human SIGLEC 3 antibody	Alzheimer’s disease	i.v.	Phase I	NCT03822208
Crenezumab	Humanized IgG4 monoclonal antibody targeting Aβ	Alzheimer’s disease	i.v.	Phase II	NCT01397578; NCT01343966; NCT01723826; NCT01998841; NCT02670083
Donanemab	Humanized IgG1 monoclonal antibody targeting N3pG- Aβ	Alzheimer’s disease	i.v.	Phase II	NCT03367403
JNJ-63733657	Monoclonal antibody targeting the mid-region of tau	Alzheimer’s disease	i.v.	Phase I	NCT03375697
Semorinemab	Anti-tau IgG4 antibody	Alzheimer’s disease	i.v.	Phase II	NCT02820896; NCT03828747; NCT03289143
Solanezumab	Humanized monoclonal IgG1 antibody	Alzheimer’s disease	i.v.	Phase III	NCT00329082; NCT00749216; NCT00904683; NCT00905372; NCT01148498; NCT01127633; NCT01760005; NCT01900665; NCT02008357; NCT02760602
Zagotenemab	Humanized anti-tau antibody	Alzheimer’s disease	i.v.	Phase II	NCT03518073
Opicinumab	Monoclonal antibody targeting LINGO1	Multiple sclerosis	i.v.	Phase II	NCT02833142; NCT03222973; NCT01721161
Rindopepimut	EGFRvIII peptide vaccine	Glioblastoma	i.d.l	Phase II	NCT01480479; NCT01498328; NCT00458601
Durvalumab	Human IgG1κ monoclonal antibody	Glioblastoma	i.v.	Phase II	NCT02336165
^125^I-MAB-425	Anti-epidermal growth factor receptor-425 monoclonal antibody	Glioblastoma	i.v. or i.a.	Phase II	NCT01317888
^131^I-chTNT-1/B MAb	Monoclonal antibody targeting DNA-histone H1 complex	Glioblastoma	s.c.	Phase II	NCT00677716; NCT00509301; NCT00128635; NCT00004017
^188^Re-labeled Nimotuzumab	Humanized monoclonal antibody targeting epidermal growth factor receptors	Glioblastoma and astrocytoma	Intracavity	Phase I	[227]
^211^At-labeled 81C6 mAb	Chimeric antitenascin monoclonal antibody	Brain tumor	Intracavity	Phase I	[228]; NCT00003461
^131^I-Omburtamab	Murine monoclonal antibody targeting 4Ig-B7-H3	Neuroblastoma and leptomeningeal metastases	i.c.v.	Phase II/III	[229]; NCT03275402

TREM2: triggering receptor expressed on Myeloid cells-2; SIGLEC: sialic acid binding Ig-like lectins; LINGO: nogo receptor-interacting protein; EGFR: epidermal growth factor receptor; i.c.v.: intracerebroventricular; s.c.: subcutaneous; i.v.: intravenous; i.d.: intradermal; i.a.: intraarterial.
